# Delayed hemodialysis in COVID-19: Case series with literature review

**DOI:** 10.5414/CNCS110240

**Published:** 2021-03-11

**Authors:** Michael Connerney, Yasar Sattar, Hiba Rauf, Sahil Mamtani, Waqas Ullah, Nara Michaelson, Umaima Dhamrah, Naman Lal, Sharaad Latchana, Aaron Saul Stern

**Affiliations:** 1 Internal Medicine, Icahn School of Medicine at Mount Sinai – Elmhurst Hospital, Queens, New York,; 2 Internal Medicine, American Society of Clinical Oncology, Alexandria, VA,; 3 Internal Medicine, Jacobi Medical Center, Bronx, NY,; 4 Internal Medicine, Abington Jefferson Hospital, PA,; 5 Neurology, New York-Presbyterian/Weill Cornell, New York, NY, USA,; 6 Avalon University School of Medicine, Curacao, and; 7 Division of Nephrology, Icahn School of Medicine at Mount Sinai – Elmhurst Hospital, Queens, NY, USA

**Keywords:** hemodialysis, ESRD, COVID-19, ACE-2, angiotensin II

## Abstract

Background: Increased incidence of kidney injury has been seen in patients with COVID-19. However, less is known about COVID-19 susceptibility and outcomes in end-stage renal disease (ESRD) patients on hemodialysis (HD). Reduced angiotensin-converting enzyme 2 (ACE-2) from SARS-CoV-2 binding and increased angiotensin II (Ang-II) activity have been suggested as mechanisms for COVID-19 renal pathophysiology. Materials and methods: In this case series, we analyzed the data of 3 patients with ESRD who had a delay in receiving their regular HD. Reduced oxygen requirement, resolved hyperkalemia, and normalized fluid status were used for the basis of discharge. Results: Presenting symptoms included fever, dyspnea, and dry cough. Laboratory markers were characteristic for COVID-19, such as lymphopenia, elevated D-dimer, C-reactive protein (CRP), and interleukin 6 (IL-6). All 3 of our reported patients required urgent HD upon admission. However, we report no fatalities in our case series, and our patients did not have a severe course of illness requiring endotracheal intubation. We reviewed COVID-19 pathophysiology and how patients with ESRD on HD may be particularly at risk for infection. Conclusion: New renal failure or ESRD sequelae, such as hyperkalemia, uremic encephalopathy, and fluid overload, can be exacerbated by a delay in receiving HD due to COVID-19 infection. Both direct COVID-19 infection and the challenges this pandemic creates to health care logistics present unique threats to ESRD patients on HD.

## Introduction 

Severe acute respiratory syndrome coronavirus-2 (SARS-CoV-2), the causative agent of coronavirus disease 2019 (COVID-19), has involved 227 countries and territories across the globe, with more than 14.6 million cases and 608,000 deaths [1]. COVID-19 incidence has skyrocketed in the US with nearly 4 million diagnosed cases and 143,000 deaths by the end of July 2020 [2]. COVID-19 pulmonary manifestations are well described in the literature, commonly presenting as a viral pneumonia with symptoms such as fever, dyspnea, dry cough, diarrhea, and abdominal pain, often preceding a course of hypoxemia that may progress to acute respiratory distress syndrome (ARDS) and mechanical ventilation [3]. Extrapulmonary manifestations of COVID-19 often involve the renal system, and this may be found in up to 25% of COVID-19 cases [4]. 

In an attempt to better understand COVID-19 renal manifestations, we have reviewed the literature on SARS-CoV-2 cell entry via angiotensin-converting enzyme 2 (ACE-2) binding and its effect on angiotensin II (Ang-II). It has been reported that ESRD patients are in a state of immunosuppression due to chronic uremia [5]. Severe uremia in new renal failure or ESRD from delayed hemodialysis (HD) may worsen this immunosuppression and increase the risk for COVID-19 morbidity and mortality. This pandemic has created multiple challenges for ESRD patients as dialysis centers pose a significant risk for COVID-19 transmission. Here we present the outcomes of three COVID-19 ESRD cases who missed their regular HD, and we will discuss the potential strategies to overcome the logistic issues faced in dialysis centers during COVID-19 pandemic. 

## Case 1 

**Figure 1:**
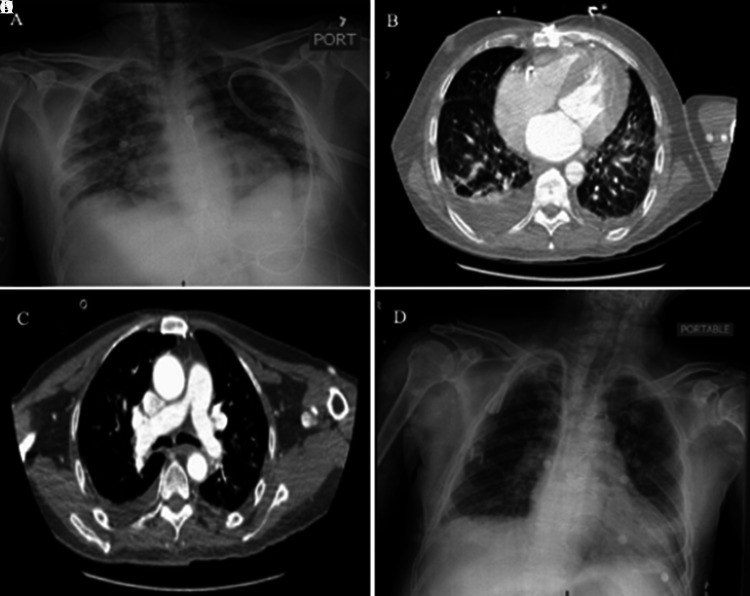
A: Chest X-ray (CXR) posteroanterior (PA) view of patient 1 showing patchy ground-glass opacities in all lung fields bilaterally. B: Chest CT-angiography of patient 2 showing diffuse ground glass opacities with micronodules and right-sided pulmonary effusion. C: Chest CT-angiography of patient 2 showing normal pulmonary arteries ruling out pulmonary embolism. D: CXR PA view of patient 3 showing diffuse, patchy ground-glass opacities in bilateral lung fields.

A 42-year-old man with a past medical history (PMH) of adult polycystic kidney disease (APKD) with end-stage renal disease (ESRD, anuric) newly on hemodialysis (HD) for 1 month presented to the emergency department (ED) with altered mental status (AMS) after missing his last 2 HD sessions as he was denied by his HD center due to suspected COVID-19 symptoms. The patient reported 6 days of subjective fever, cough, dyspnea, and malaise. He denied chest pain, nausea, vomiting, abdominal pain, diarrhea, sick contacts at home, or travel. The patient denied any history of smoking or the use of any illicit drugs. Initial vitals were temperature (T) of 38.9 °C, respiratory rate (RR) 30 breaths per minute (BPM), oxygen saturation (O_2_ sat) 93% on room air, heart rate (HR) 95 beats per minute (BPM), and blood pressure (BP) 138/74 mmHg. Pertinent exam findings were disorientation to time and place, bilateral crackles on lung exam, and no leg edema. Laboratory results are shown in Table 1. Chest X-ray (CXR) showed diffuse bilateral ground glass opacities (GGOs) more pronounced on the peripheral lung fields (Figure 1A). Electrocardiogram (EKG) showed no acute ischemic changes or hyperkalemic changes. A real-time reverse transcriptase-polymerase chain reaction (RT-PCR) nasal swab for SARS-CoV-2 returned positive. 

Given the viral symptoms, elevated inflammatory markers, positive swab, and uremia with high anion gap metabolic acidosis (HAGMA), the patient was diagnosed with COVID-19 and uremia in the setting of missing HD. The patient was started on a 5-day regimen of hydroxychloroquine (400 mg b.i.d. on day 1 then 400 mg daily), and azithromycin (500 mg daily). The patient also received coverage for community-acquired pneumonia with 5-day regimen of ceftriaxone given his leukocytosis, but it was less concerning given negative blood and sputum cultures. Emergent HD was started using his left upper extremity arteriovenous fistula with net ultrafiltrate (UF) removal to maintain net negative 2 – 3 L. The patient’s oxygen requirement improved from initial 2 L O_2_ via nasal cannula (NC) to a normal O_2_ sat on room air. His inflammatory markers down-trended, clinical symptoms including altered mental status resolved to normal, and he was discharged home with outpatient HD arranged and home quarantine precautions for the following 2 weeks. 

## Case 2 

A 70-year-old man with a PMH of ESRD secondary to hypertension (HTN), coronary artery disease (CAD), coronary artery bypass grafting (CABG) in 2011, and type 2 diabetes mellitus (DM2) presented to the ED with chest pain, dyspnea, dry cough, fatigue, and frank hemoptysis for 2 days. He denied nausea, vomiting, abdominal pain, or diarrhea. The patient was on HD for 2 years and 3 months and missed 2 sessions of HD due to transportation issues during the COVID-19 lockdown. The patient denied smoking or using any illicit drugs. Admission vitals were T: 39.4 °C, RR: 16 BPM, O_2_: sat 81% on RA, HR: 68 BPM, BP: 257/113 mmHg. Physical exam revealed a cachectic, ill-appearing male with diffuse bilateral rales on auscultation of lungs. Laboratory results are shown in Table 1. EKG showed no signs of ischemia or hyperkalemic changes. CT angiogram was performed to rule out the broad differential diagnosis of hemoptysis including pulmonary embolism, and it showed diffuse GGOs without any pulmonary emboli (Figure 1B, C). A COVID-19 nasal swab RT PCR was positive. 

Given the patient’s respiratory symptoms, uremia with hypertensive urgency, and positive swab patient was diagnosed with COVID-19 and ESRD-induced hypertensive urgency, platelet dysfunction, and demand ischemia. The patient was treated with the same hydroxychloroquine and azithromycin regimen as with case 1. For hypertensive urgency, the patient initially received IV labetalol pushes of 20 mg × 2. He was started on urgent HD using his left upper extremity arteriovenous fistula with a net UF removal of 4.2 L. His clinical symptoms, vital signs, and inflammatory markers resolved to normal range. Overall oxygen requirement improved from initial continuous positive airway pressure (CPAP) 100% fractional inspired O_2_ at 8 cm H_2_O pressure to a normal O_2_ sat on room air. He was discharged with outpatient HD follow-up and instructions for home quarantine for an additional 2 weeks once discharged. 

## Case 3 

A 66-year-old man with a PMH of ESRD secondary to DM, CAD, congestive heart failure (CHF) with ejection fraction of 20%, HTN, and DM2 with diabetic retinopathy, presented to the ED with dry cough, dyspnea, fatigue, and diarrhea for 2 days. The patient had been receiving HD for the past 8 months. He missed 1 HD session as the outpatient dialysis facility denied his appointment due to the suspected symptoms of COVID-19. He denied nausea, vomiting, and abdominal pain. The patient also states he is a former smoker with a history of 60 pack-/years. He denied the use of any illicit drugs. Vitals at presentation were T: 39.2 °C, RR: 18 BPM, O_2_ sat: 91% on room air, HR: 107 bpm, BP: 180/66 mmHg. Physical exam revealed an ill-appearing male with diffuse bilateral rales on auscultation of lungs. Pertinent laboratory results are shown in Table 1. Chest X-ray (CXR) showed diffuse patchy infiltrates and GGOs (Figure 1D). EKG showed no indications of acute ischemic cardiomyopathy or electrolyte changes. A COVID-19 nasal swab for real-time reverse transcriptase-polymerase chain reaction (RT-PCR) returned positive. 

Given the patient’s viral respiratory symptoms, clinical markers, and positive swab RT-PCR, the patient was diagnosed as COVID-19 with viral enteritis as well as uremia in the setting of missed HD. The patient was treated by a similar regimen for COVID-19 as in cases 1 and 2. The patient started on urgent HD using his right subclavian double lumen HD catheter, and received 2 sessions in the hospital, with each session having 3 L of UF removed. His oxygen requirement improved from an initial 2 L O_2_ via NC to a normal O_2_ sat on room air. 

His clinical symptoms and inflammatory markers improved status post treatment. As with cases 1 and 2, care was made to arrange for safe COVID-19 outpatient HD follow-up, and he was ultimately discharged with instructions for home quarantine for 2 additional weeks. 

## Discussion 

Given the ongoing COVID-19 pandemic and efforts to decrease facility transmission, it is not uncommon to see missed HD at outpatient centers with patients being transferred to hospitals for COVID-19 rule-out. Not only does a delay in HD put patients at risk for worsening uremia symptoms and hyperkalemia, but it may also worsen COVID symptoms. CKD and ESRD can increase overall risk of primary infection or superimposed bacterial infection leading to sepsis [5, 6]. Missing HD worsens chronic uremia, which in turn may cause immunosuppression with T-cell and platelet dysfunction [7]. Furthermore, premature lymphocyte cell aging and dysfunction has also been found in ESRD with baseline uremia [8]. It is possible that ESRD with uremia exacerbates the profound lymphopenia and platelet dysfunction known to be associated with COVID-19, leading to the mild hemoptysis in our case 2. 

ESRD and HD with worsening uremia in the setting of missed HD increases overall infectious susceptibility, which may include increased susceptibility to COVID-19 [5]. Another consideration for high COVID-19 susceptibility may be in the setting of chronic renin-angiotensin-system (RAS) dysregulation from reduced baseline ACE-2 in ESRD patients [9]. ACE-2, a cell-surface receptor protein, is present in arterial and venous endothelial cells as well as in the lungs, heart, intestines, and kidneys [10]. A vital role of ACE-2 within the RAS system is its cleaving action on Ang-II, a vasoconstrictor, into angiotensin 1-7, which are vasodilator molecules. SARS-CoV-2 was recently shown to bind to ACE-2 receptors for receptor-mediated endocytosis during cell infection using the TMPRSS-2 protein, which downregulates ACE-2 at the cell surface [11]. COVID-19 can be associated with AKI, CKD, and renal failure, and signs of renal injury with COVID-19 are linked with high mortality outcomes [12]. The reduced ACE-2 in ESRD can be linked to increased vascular permeability, worsening pulmonary edema, and worsening ARDS, also known as nephrogenic ARDS in the setting of cytokine storm [13]. High Ang-II levels in ESRD and COVID-19 is associated with worsening of hypertension in the setting of vasoconstrictive effects as seen in case 2 [14]. 

During the COVID-19 pandemic, given the quarantine, home isolation, and scarcity of COVID specific HD centers, COVID-19 symptoms at the time of dialysis can be a factor for missed HD sessions in the outpatient setting. Furthermore, even urgent HD in the hospital setting is prolonged due to the COVID-19 overburdening of staff and resources, including delay in ambulance transport, delay in ED triage, time for use of personal protective equipment (PPE) by each individual for every interaction with the patient, delayed transport of COVID cases given low bed availability, delay in PPE use by transporters, or delay in COVID-19 designated HD machines in the setting of precautionary measures. Due to the myriad of factors that can cause delay, patients are presenting with ESRD sequelae that include uremic encephalopathy, platelet dysfunction, hypertensive crisis, hyperkalemia, and immunosuppression as seen in our cases. These challenges can be mitigated by the opening of COVID-specific outpatient HD centers and inpatient COVID specific HD units. 

There exist multiple logistic issues in treating and preventing the cluster of COVID infection in the dialysis facilities. These challenges include cost of screening, lack of isolated rooms, scarcity of PPE, medical staff logistics, and inadequate health education among patients and caregivers. Similar issues were experienced by our 3 patients who missed multiple sessions of HD due to insufficient outpatient facilities and transportation. The International Society of Nephrology (ISN) has recommended universal screening for all dialysis patients. However, it might be difficult to perform this in resource-challenged areas. In such cases, centers should at the very least screen symptomatic patients [15]. Providers should have a low threshold for screening as patients with ESRD may present with atypical symptoms like gastrointestinal complaints and loss of taste/smell [16]. The real-time reverse transcriptase-PCR (RT-PCR) is the gold standard test for early diagnosis due to its high sensitivity, specificity, and fast detection in 3 hours [16]. The fast testing can assist the clinicians in providing timely management and transfer to a COVID-19-specific dialysis center. The variations of viral RNA sequences in the primer and probe target region of SARS-CoV-2 due to rapid evolution and mutations may produce false negative results. As the accuracy of this test is not 100%, screening should be combined with clinical features and imaging to facilitate the diagnosis of COVID-19 infection [17]. Other tests, such as point-of-care tests and rapid antigen tests, can be performed within 5 – 10 minutes, but have high rates of false negatives, which can compromise patient safety and promote viral transmission [18, 19]. 

Communication barriers between medical staff and patients may lead to undiagnosed symptoms or missed HD sessions. Therefore, medical personnel should contact HD patients before appointments to identify any suspicious symptoms or instruct them to call back to report any unusual findings. However, it is the responsibility of HD providers that patients receive their scheduled HD regardless of COVID-19 infection status, and such coordination should not be left entirely to the patients. COVID-19-positive patients should be isolated and receive dialysis treatment in a private dialysis room with air-borne protection. In the absence of an air-borne isolation room, a private room should be assigned, and if that is also not available then the patient should receive treatment 6 feet away from other patients in a corner ventilated area of the center. The mild to moderate COVID cases should receive HD in an out-patient setting whereas severe patients should be transferred to in-patient dialysis units. It is crucial to maintain the safety of medical staff as an outbreak in the staff can disrupt an entire dialysis center. Li et al. [16] has recommended grouping of staff in different shifts and working areas to prevent contact between them. The transportation of patients from home to dialysis center is a challenge, but this vital aspect of HD coordination will require collaboration with all available transportation organizations. In these circumstances, the use of personal protective equipment (PPE) including N-9 5 masks, gloves, gowns, and eye protection must be emphasized, with special attention to disinfection [20]. Furthermore, the gaps in health education can be bridged through conducting remote or onsite information sessions regarding the correct use of PPE, hand hygiene, and proper disposal of contaminated items. At Elmhurst Hospital Center in New York, we have both inpatient and outpatient COVID-19-specific HD centers where COVID-19-positive ESRD patients can attain expedited HD, as was received by our presented cases. Contingencies should be undertaken by hospitals and HD centers to adequately meet the regular HD needs of COVID-19-positive ESRD patients. 

## Conclusion 

ESRD patients on HD represent a group of people who are uniquely positioned to be adversely affected by COVID-19. From inherent increased infection risk, RAS dysregulation via decreased ACE-2, and difficulties with safely coordinating HD logistics, patients with ESRD will require more attention in the setting of COVID-19. We believe our case series highlights several issues that may be encountered by future providers treating COVID-19-positive ESRD patients. Creating COVID-19-specific HD units for both the inpatient and outpatient settings is an important step in mitigating the delay of timely HD during this pandemic. Nevertheless, the logistic issues in dialysis facilities during the COVID-19 pandemic may interfere with the treatment of ESRD. The strategic utilization of resources and adhering to guidelines is critical in overcoming these challenges. 

## Learning points

- Missed HD sessions during COVID-19 pandemic can present uremic or hypertensive sequela.
- ACE-2/Ang-II imbalance may be implicated in the AKI and acute renal failure (ARF) seen in COVID-19. COVID-19 may exacerbate the baseline ACE-2/Ang-II imbalance seen in ESRD that could worsen COVID-19 infection outcomes due to further RAS dysregulation.
- Missing regular HD appointments, or delay in HD, has been a common presentation during the COVID-19 pandemic. COVID-19-specific HD centers inpatient and outpatient are helpful to expedite care to avoid preventable complications.
- The systematic guidelines from different nephrology societies should be followed to counter the logistic issues arising in dialysis facilities secondary to COVID-19 pandemic.

## Acknowledgment 

We would like to acknowledge our Director of Medicine, Dr. Joseph Lieber, and Program Director Dr. Lawrence Reich for their efforts in improving health care reforms and research availability at Elmhurst Hospital, New York. 

## Statement of ethics 

We present a case series consisting of 3 patients. Written informed consent was obtained by all patients involved. As this was a small case series of 3 patients, ethics approval by our Institutional Review Board (IRB) was not required. 

## Funding 

The authors have no funding sources to report. 

## Conflict of interest 

The authors report no financial relationship or conflict of interest regarding the content herein. 

**Table 1.** Pertinent laboratory values across 3 patients with COVID-19 and ESRD.

Laboratory values	Patient 1	Patient 2	Patient 3
White blood cell count, × 10^9^ /L	15.8	6.8	9.4
Platelets, × 10^9^ /L	178	101	123
Lymphocyte count, × 10^9^ /L	0.18	0.79	1.03
Neutrophil %	94.8	74.5	81.1
Lymphocyte %	18	15.2	14
Potassium, mmol/L	6	5.3	5.6
Blood urea nitrogen, mg/dL	151	18	92
Creatinine, mg/dL	16.4	11.3	14.4
Bicarbonate, mmol/L	6	18	11
Anion gap, mmol/L	33	29	33
Alanine aminotransferase (ALT), U/L	102	16	113
Aspartate aminotransferase (AST), U/L	73	28	146
High sensitivity C-reactive protein, mg/L	266	87.6	61.1
Lactate dehydrogenase, U/L	566	505	476
Procalcitonin, ng/mL	11.7	2.75	15.74
D-dimer, ng/mL	1,395	338	1,094
Interleukin-6 (IL-6), pg/mL	329	108	411
Troponin-T, ng/mL	0.065	0.238	0.163
Venous pH	7.06	7.28	7.31
Venous pCO_2_, mmHg	23	53	35
Venous pO_2_, mmHg	38	< 20	52.7

ESRD = end-stage renal disease; COVID-19 = novel coronavirus disease 2019; pCO_2_ = partial pressure of carbon dioxide; pO_2_ = partial pressure of oxygen; % = percentage; ng/mL = nanogram per milliliter; pg/mL = picograms per milliliter; mg/mL = milligram per milliliter; mmol/L = millimolar per liter; U/L = units per liter.
